# Translational Implication of Galectin-9 in the Pathogenesis and Treatment of Viral Infection

**DOI:** 10.3390/ijms18102108

**Published:** 2017-10-08

**Authors:** Jenn-Haung Lai, Shue-Fen Luo, Mei-Yi Wang, Ling-Jun Ho

**Affiliations:** 1 Division of Allergy, Immunology, and Rheumatology, Department of Internal Medicine, Chang Gung Memorial Hospital, Chang Gung University, Tao-Yuan 33305, Taiwan; lsf00076@adm.cgmh.org.tw (S.-F.L.); meiyiwang800106@gmail.com (M.-Y.W.); 2 Graduate Institute of Medical Science, National Defense Medical Center, Taipei 11490, Taiwan; 3 Institute of Cellular and System Medicine, National Health Research Institute, Zhunan 35053, Taiwan

**Keywords:** translational medicine, galectin-9, pathogenesis, treatment, viral infection

## Abstract

The interaction between galectin-9 and its receptor, Tim-3, triggers a series of signaling events that regulate immune responses. The expression of galectin-9 has been shown to be increased in a variety of target cells of many different viruses, such as hepatitis C virus (HCV), hepatitis B virus (HBV), herpes simplex virus (HSV), influenza virus, dengue virus (DENV), and human immunodeficiency virus (HIV). This enhanced expression of galectin-9 following viral infection promotes significant changes in the behaviors of the virus-infected cells, and the resulting events tightly correlate with the immunopathogenesis of the viral disease. Because the human immune response to different viral infections can vary, and the lack of appropriate treatment can have potentially fatal consequences, understanding the implications of galectin-9 is crucial for developing better methods for monitoring and treating viral infections. This review seeks to address how we can apply the current understanding of galectin-9 function to better understand the pathogenesis of viral infection and better treat viral diseases.

## 1. Introduction of Galectin-9

Galectins, as part of a group of pattern recognition receptors, are highly conserved lectins and are widely expressed in different tissues cells and immune cells [1]. The characteristic structure of galectins includes carbohydrate recognition domains of approximately 130 amino acids in length that bind β-galactosides [1]. To date, 15 galectins have been identified and were found to contain various numbers of carbohydrate recognition domains and associated structures [2]. Galectin-9 was first cloned and characterized as a T cell-derived eosinophil-specific chemoattractant, and was found to be involved in various cellular processes such as differentiation, aggregation, adhesion, and death, as well as various phases of immune responses [3,4,5]. Galectin-9 contains two distinct carbohydrate recognition domains connected by a linker peptide and is a natural ligand for the T cell immunoglobulin domain and mucin domain protein 3 (Tim-3) [6]. Previous studies revealed the increased expression of galectin-9 in different target cells in response to various stimuli, such as mitogens, agonists of toll-like receptors, and pro-inflammatory cytokines like interferon-γ (IFN-γ) and interleukin-1β (IL-1β) [5,7]. Interestingly, stimulation with interferon-γ, but not with IL-1β or lipopolysaccharide, effectively induces galectin-9 expression in macrophages derived from macrophage colony-stimulating factor-treated CD14^+^ primary monocytes [8]. In contrast, the intracellular signals that regulate galectin-9 expression are largely unknown. A previous study showed that stimulating phorbol 12-myristate 13-acetate results in the upregulation of galectin-9 expression and that this effect can be partially blocked by treatment with inhibitors of either protein kinase C inhibitor or matrix metalloproteinase [9]. Aside from endogenous and exogenous regulatory factors, the differentiation process from monocyte to macrophage can also induce the production of galectin-9 [7]. However, galectin-9 may play a role in inducing the maturation of monocyte-derived dendritic cells [10]. Harwood et al. demonstrated that maximal galectin-9 induction in monocytes requires direct contact with, or proximity to, the interacting cells [7]. 

Many signals can induce the expression and production of galectin-9. As such, the induced expression of galectin-9 has been widely demonstrated in infections caused by several different viruses, including hepatitis C virus (HCV) [8,11,12], hepatitis B virus (HBV) [13], herpes simplex virus (HSV) [14,15], influenza virus [16], Epstein-Barr virus [17], dengue virus (DENV) [18,19], and human immunodeficiency virus (HIV) [20]. Although the level varies, the increased expression or production of galectin-9 can be observed following the infection of a variety of tissue cells and immune effector cells. Li et al. reported the variable expression levels of galectin-9 on different subsets of antigen-presenting cells, such as Kupffer cells, myeloid dendritic cells, and plasmacytoid dendritic cells in patients with HBV-associated hepatocellular carcinoma (HCC), with the highest expression observed in Kupffer cells [13]. In addition, CD14^low/high^CD16^+^ monocytes produce higher amounts of galectin-9 when compared to classic CD14^+^CD16^−^ monocytes, which are associated with the severity of liver injury and fibrosis in patients with chronic HBV infection [7,21]. The enhanced expression of galectin-9 promotes significant changes in behaviors of the virus-infected cells, and the resulting events may tightly correlate with immunopathogenic processes of the viral disease. As anticipated, the variable expression of galectin-9 in different populations of immune effector cells may lead to different disease pathogenesis. The galectin-9-mediated effects can be even more complex given that galectin-9 exists and functions both intracellularly and extracellularly to regulate inflammatory responses [6].

Tim-3 is the most well-known binding receptor for galectin-9, and its expression is upregulated on the surface of immune effector cells such as CD4^+^ and CD8^+^ T cells in patients with HIV infection [22,23]. A mutual regulation of galectin-9 and Tim-3 has been reported: the knockdown of galectin-9 downregulates Tim-3 levels, and the knockdown of Tim-3 inhibits galectin-9 secretion in U-937 cells [24]. However, previous studies suggest the presence of Tim-3-independent galectin-9-mediated mechanisms [25,26], and thus, Tim-3 may not be the only receptor for galectin-9. Intriguingly, given that the secretion of galectin-9 is dependent on Tim-3 in human acute myeloid leukemia cells, this Tim-3-independent mechanism does not exist in lymphoid cells [24]. In contrast, the galectin-9 ligation-induced changes of many transcriptional and functional genes are independent of Tim-3 in natural killer (NK) cells [12]. Clayton et al. reported that galectin-9 binds to multiple immune accessory molecules, such as the receptor phosphatases CD45 and CD148; moreover, by binding to these two molecules, galectin-9 enhances their interaction with Tim-3 within the CD3 signaling complexes [27]. Galectin-9 binds to CD44 and blocks the interaction between CD44 and hyaluronan, which consequently inhibits airway inflammation and airway hyperresponsiveness [28]. Program death-1 (PD-1), rather than Tim-3, is more crucial for galectin-9 to regulate T cell function and migration in HIV infection [29]. Furthermore, treatment with galectin-9 induces apoptosis in plasma cells purified from MRL/lpr lupus-prone mice, and the effect cannot be prevented with anti-Tim-3 monoclonal antibodies (mAb); this suggests the involvement of a non-Tim-3 receptor for galectin-9-mediated apoptosis in plasma cells [30]. Lhuillier et al. also observed that galectin-9 induces apoptosis of Jurkat T cells in a Tim-3-independent manner [31]. Biochemical and biophysical studies on Tim-3 revealed a unique structural feature that is conserved in TIM family members that may mediate a galectin-9-independent binding process [32].

In addition to galectin-9, other molecules may serve as Tim-3 ligands. According to Nakayama et al., Tim-3–immunoglobulin fusion protein can weakly but substantially bind to phosphatidylserine, but not to phosphatidylethanolamine, phosphatidylinositol, or phosphatidylcholine on apoptotic cells through an FG loop in the immunoglobulin-like variable (IgV) domain [33]. Tim-3 binds to carcinoembryonic antigen-related cell adhesion molecule 1 to induce T cell tolerance and exhaustion [34]. Given that Tim-3 suppresses innate responses to nucleic acids, the mechanisms appear to involve the interaction between Tim-3 and alarmin HMGB1, which inhibits the recruitment of nucleic acids into the endosomes of dendritic cells [35].

TIM family proteins play roles in enhancing the entry of a wide range of enveloped viruses through the interaction between the IgV domain of TIM proteins and the virion-associated phosphatidylserine [36]. However, not all TIM family members effectively promote viral entry; Tim-3 contains an IgV domain that binds phosphatidylserine, but it cannot effectively enhance viral entry. Overexpression of human Tim-3 causes HeLa cells to become partially susceptible to hepatitis A viral (HAV) infection [37]. In addition, in sharp contrast to the overexpression of Tim-1 or Tim-4, overexpression of Tim-3 in 293T cells only mildly increases the percentage of DENV-infected cells [38]. Many factors may determine the binding avidity between lectins and glycans, such as the structure and the density of glycan epitopes on glycoproteins and their cell surface density [39]. Accordingly, the interaction between different TIM proteins and viruses provides a good example to explain this phenomenon.

## 2. Galectin-9/Tim-3 Interaction Regulates the Immune Response

**Figure 1:**
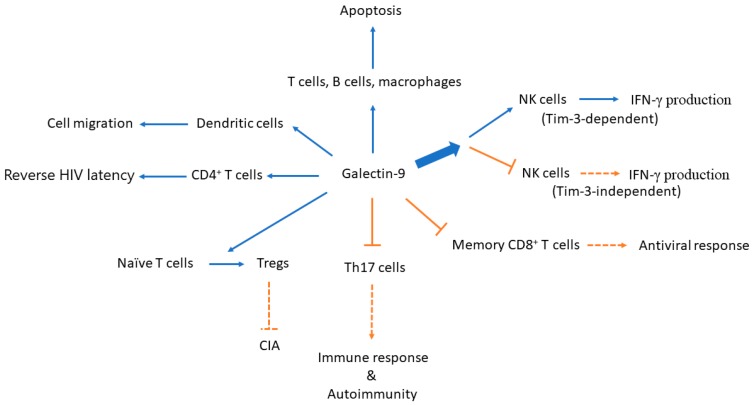
A simplified diagram illustrates the effects of galectin-9 on immune cells and the potential consequences. Expression of a proteolysis-resistant galectin-9 construct induces apoptosis in immune cells as well as in malignant cells. Galectin-9 causes the differentiation of naive T cells to Tregs and inhibits differentiation towards T helper (Th)17 cells. Both effects lead to the inhibition of the development of immune response and autoimmune arthritis in a CIA animal model. While treatment with recombinant human galectin-9 induces IFN-γ production in a Tim-3-overexpressing NK cell line and in Tim-3^+^ primary NK cells induced with low-dose IL-12 and IL-18, galectin-9 ligation has also been shown to reduce the proportion of IFN-γ-producing NK cells stimulated with IL-12/IL-15 in a Tim-3-independent manner. Galectin-9 treatment extensively regulates gene expression of molecules involved in the processes of human immunodeficiency virus (HIV) latency. Galectin-9 may also regulate dendritic cell migration in the example of dengue virus (DENV) infection. NK, natural killer; CIA, collagen-induced arthritis; IFN-γ, interferon-γ; and, Tregs, regulatory T cells. Blue arrow: induce; bold blue arrow: simply means there are two diverging effects out of this direction; orange T-bar arrow/orange dotted T-bar arrow: inhibit; orange arrow with dotted line: reduce.

Homeostasis of the immune system requires both positive and negative machineries to tightly control various phases of cellular interaction or cell-environment communication. The binding between galectins and glycans leads to sequential signaling events and plays important roles in immune tolerance and inflammation [40]. Depending on the specific model or target examined, galectin-9 may either induce or suppress inflammation [41,42]. Expression of a proteolysis-resistant galectin-9 construct induces apoptosis in hematological, dermatological, and gastrointestinal malignant cells [43] (Figure 1). When compared to control mice, galectin-9-deficient mice are more susceptible to developing collagen-induced arthritis, which is reflected by an increase in the numbers of CD4^+^ Tim-3^+^ T cells and a decrease in the numbers of Foxp3^+^ regulatory T cells (Tregs) in these mice. In vitro treatment of naïve T cells with galectin-9 promotes differentiation into Tregs and inhibits differentiation into T helper (Th)17 cells [44]. In addition, the interaction between galectin-9 and Tim-3 inhibits T cell proliferation and triggers T cell apoptosis [22]. Moreover, treatment with recombinant human galectin-9 inhibits the release of Th1/Th2/Th17 cytokines in co-cultures of autologous monocyte-derived dendritic cells and peripheral blood lymphocytes from healthy controls and from patients with autoimmune thyroid disease [45]. The negative correlation between galectin-9 mRNA levels in peripheral blood mononuclear cells of patients with rheumatoid arthritis and the disease activity provides additional evidence that galectin-9 is anti-inflammatory [46]. A similar conclusion was also reached in a xenograft-interaction model. A co-culture of human galectin-9-expressing porcine kidney epithelial cells and M1-differentiated THP-1 cells suppresses the production of pro-inflammatory cytokines and reduces the cytotoxic effects of the cells [47]. Together, these results from different studies suggest that galectin-9-mediated signaling events may lead to the inhibition of inflammatory responses [48,49].

In contrast, some results suggest that galectin-9 may mediate pro-inflammatory responses. Galectin-9 was originally found to be produced by T cells to serve as an eosinophil chemoattractant that mediates pro-inflammatory responses [3,4,5]. The concentrations of galectin-9 produced by activated astrocytes in cerebrospinal fluid positively correlate with the number of lesions on T1 weighted images of MRI, but not with gadolinium enhancing lesions, in patients with secondary progressive multiple sclerosis [50]. Treatment with recombinant human galectin-9 induces the production of IFN-γ in a Tim-3-overexpressing NK cell line, and in Tim-3^+^ primary NK cells induced with low-dose IL-12 and IL-18 [51]. However, galectin-9 ligation has also been shown to reduce the proportion of IFN-γ-producing NK cells stimulated with IL-12/IL-15, downregulate NK cell-mediated cytotoxicity, and inhibit lymphokine-activated killing. Interestingly, these observed effects appear to be independent of Tim-3 [12], and together, these studies suggest that the effects of galectin-9 may differ depending on the interacting molecules. Furthermore, galectin-9 induces T helper cells to produce pro-inflammatory cytokines in a dose-dependent Tim-3-independent manner [25]. By enhancing IFN-γ-induced pro-inflammatory effects, galectin-9 potently mediates neuroinflammation in the mouse hippocampus [52].

Structural biology studies have investigated the differential roles of galectin-9-mediated immune responses. Crystal or X-ray structural analysis of both the N- and C-terminal carbohydrate recognition domains of galectin-9 has been conducted [53,54]. The studies indicate that the N-terminal and the C-terminal carbohydrate recognition domains have different binding specificities in recognizing branched and α 2-3-sialylated oligosaccharides [54]. These differences in binding specificity were further investigated by generating various mutant, wild-type, or homodimer constructs containing N- or C-terminal regions of the carbohydrate recognition domains of galectin-9 [55]. The results showed that all of the examined galectin-9 constructs differentially regulate dendritic cell activation and T cell death: the C-terminal carbohydrate recognition domain is more potent in inducing T cell death, and the N-terminal carbohydrate recognition domain is more effective in activating dendritic cells to induce the production of pro-inflammatory cytokines. According to computer analysis, these constructs also preserve different patterns and affinities in binding to Tim-3 [55]. 

## 3. Galectin-9 in Viral Pathogenesis

The effects of galectin-9 greatly affect the immune defense mechanisms against viral infection. Some of the galectin-9-mediated effects have been investigated in several examples of viral infection. Depending on the target cells and the mechanisms of disease pathogenesis in individual viral infections examined, the roles of galectin-9 in viral pathogenesis may moderately differ. Below, we will discuss some reported features of galectin-9/Tim-3 in the immunopathogenesis of different viral infections.

A previous study found that the reduction in the numbers of CD4^+^ T helper cells and the impaired virus-specific CD8^+^ T cell response are both major factors responsible for persistent infection in HCV-infected patients [56]. To identify factors contributing to the impaired virus-specific CD8^+^ T cell response, the authors examined the expression of two negative regulatory receptors, namely, Tim-3 and PD-1, on cytotoxic T cells (CTLs) in HCV-infected patients. The results showed that the population of PD-1^−^Tim-3^−^ HCV-specific CTLs significantly outnumbers the population of PD-1^+^Tim-3^+^ CTLs in patients with acute resolving infection [11]. Moreover, the population of PD-1^+^Tim-3^+^ T cells are enriched within the central memory T cell subset and within the liver [11]. While inhibiting either PD-1 or Tim-3 similarly results in the increased proliferation of HCV-specific CTLs, only the suppression of Tim-3 increases cytotoxicity against a hepatocyte cell line expressing cognate HCV epitopes [11]. Kared et al. also demonstrated that, during acute HCV infection, the progression to persistent infection is associated with increased plasma levels of galectin-9 and the expansion of galectin-9-expressing Tregs [57].

A co-culture of purified healthy CD4^+^ T cells and HCV-infected hepatocytes, which express higher levels of galectin-9 and transforming growth factor-β than that of control cells, promotes CD25^+^Foxp3^+^ Tregs differentiation [58] in a Tim-3-dependent manner. However, galectin-9 induces apoptosis of HCV-specific CTLs [8]. In addition, co-incubation with HCV-infected hepatocytes also induces apoptosis of CD4^+^CD25^+^Foxp3^–^ effector T cells [58]. Moreover, the interaction between Tim-3 on the surface of virus-specific CD8^+^ T cells and galectin-9 on Kupffer cells may induce apoptosis of T cells infiltrating the HCV-infected liver [59]. The work from these studies suggests that galectin-9 negatively impacts the immune response, thus causing liver damage during HCV infection. Nevertheless, according to Mengshol et al., treating hepatic and peripheral blood mononuclear cells with galectin-9 induces the production of pro-inflammatory cytokines, including IL-1β, TNF-α, and IFN-γ; however, galectin-9 also induces anti-inflammatory cytokines, such as IL-4, IL-10, and IL-13, in peripheral mononuclear cells [8].

In response to HSV infection, when compared to the wild-type animals, galectin-9-deficient mice have increased the frequencies and numbers of IFN-γ^+^TNF-α^+^ T cells suggesting stronger virus-specific CD8^+^ T cell responses [14]. In addition, the galectin-9 knockout mice but not the control mice developed sustained virus-specific CD8^+^ memory T cell responses [14]. Administering α-lactose to HSV-infected animals to block galectin-9 not only reproduces the effects observed in galectin-9 knockout, but also diminishes Tregs responses in animals [14]. In contrast, intraperitoneal injection of galectin-9 reduces the HSV-specific CD8^+^ T cell response and delays viral clearance [14]. In a different animal model, HSV-infected galectin-9 knockout mice have reduced numbers of Tregs in trigeminal ganglion as compared to the control animals [15]. In contrast, the galectin-9 knockout mice have stronger CD8^+^ T cell responses in trigeminal ganglion [15]. Although spontaneous reactivation occurs equally well in ex vivo trigeminal ganglion cultures of both wild-type and galectin-9 knockout mice, the reactivation was delayed in galectin-9 knockout mice when compared to wild-type mice [15].

A previous study revealed that, compared to cells in pre-reactivation status, there is a significant increase in galectin-9 transcription in the peripheral blood mononuclear cells of transplant recipients with post-reactivation of human cytomegalovirus infection [60]. Additionally, this study did not find any significant change in the expression of galectin-9 in transplant recipients without reactivation of viral infection [60], but did find that IFN-β is likely to play roles in mediating this effect [60]. Cytomegalovirus infection also induces higher production of IFN-γ from hepatic NK cells in galectin-9 knockout mice than in control mice [12]. Interestingly, the mechanisms involved in cytomegalovirus infection are reminiscent of the influenza A virus infection, such that galectin-9 knockout mice have a stronger virus-specific CD8^+^ T cell response that develops during the acute phase than do control mice [16]. In addition, the plasma levels of virus-specific immunoglobulins, including IgM, IgG, and IgA, increase in combination with enhanced Ab-secreting B220^int^ CD138^+^ cells [16]. Furthermore, better viral clearance is observed in galectin-9 knockout mice than in wild-type mice [16]. 

Of note, in addition to regulating immunocompetency and apoptosis of T cells, there have been recent publications that have comprehensively examined galectin-9 function. In one study delineating the mechanisms of establishing HIV latency, recombinant galectin-9 treatment extensively regulates the gene expression of molecules involved in key transcription initiation, chromatin remodeling, and promoter-proximal pausing processes, which are critical in the processes of HIV latency [61]. In addition, the interaction between galectin-9/Tim-3 signaling and follicular CD4^+^ T helper cells also plays a role mediating the persistence of chronic HCV infection [62]. Galectin-9 may have a role in regulating dendritic cell migration. A recent study found that galectin-9 knockdown impairs DENV-induced dendritic cell migration towards the chemoattractants CCL19 and CCL21, and that the receptor of these two chemokines CCR7 was not affected [63]. This suggests that defective migration of galectin-9-deficient cells may be caused by the inhibition of CCR7-mediated downstream signaling [63].

## 4. Galectin-9/Tim-3 Signaling-Related Markers Predict the Severity of Viral Infection and Prognosis

A population of HBV-infected patients may progress to develop HCC. Blocking the galectin-9/Tim-3 signaling pathway increases the proliferation and cytokine production from tumor-infiltrating Tim-3^+^ T cells [13]. Importantly, the numbers of Tim-3^+^ tumor-infiltrating cells are negatively associated with survival in patients with HBV-associated HCC [13]. The study raises an interesting observation that the galectin-9/Tim-3 expression may serve as a useful prognostic marker in patients with HBV-associated HCC [13].

The increased expression of galectin-9 in peripheral blood mononuclear cells positively correlates with viremia and negatively correlates with CD4^+^ T cell counts in patients with HIV infection [20]. However, although the loss of Tim-3^+^ NK cells over time is more pronounced in HIV-infected patients with low CD4^+^ T cell counts, there is no difference in plasma galectin-9 levels between HAART (highly active antiretroviral therapy)-treated and untreated subjects with progressive or controlled HIV infection [20]. In contrast, Tandon et al. found that the elevation of plasma galectin-9 is not only detected in the acute stage of HIV infection, but is also detected in chronic HIV-infected patients during suppressive antiretroviral therapy and in a select number of control patients [64]. Statistical analysis revealed that plasma galectin-9 levels not only correlate with viral RNA loads in CD4^+^ T cells in HIV-infected individuals, but also associate with the quantity and binding avidity of circulating anti-HIV antibodies [61]. In addition, the plasma levels of Tim-3 increase in patients with early and chronic untreated HIV infection [65]. Furthermore, plasma Tim-3 levels and the frequency of Tim-3-expressing plasmacytoid dendritic cells in patients with HIV infection were found to positively correlate with viral load and to negatively correlate with CD4^+^ T cell counts [65,66]. Tim-3-positive plasmacytoid dendritic cells that produce IFN-α or TNF-α in response to TLR7 or TLR9 agonist stimulation were identified infrequently [66]. Thus, Tim-3 can serve as a useful biomarker of dysfunctional plasmacytoid dendritic cells in HIV-infected patients.

The serum levels of galectin-9 but not galectin-1 or galectin-3 increase in DENV-infected patients [18]. However, there is no significant correlation between serum levels of galectin-9 and the white blood cell or platelet count [18]. Studies from another group of researchers demonstrated that serum galectin-9 levels are much higher in patients with dengue hemorrhagic fever than in patients with dengue fever only [67]. In these studies, stepwise discriminative analysis on serum levels of multiple cytokines and chemokines was then undertaken to determine the possible differences between dengue hemorrhagic fever patients and dengue fever patients. The results showed that the combined analysis of several markers, including eotaxin, galectin-9, IFN-α2, and monocyte chemoattractant protein-1, can help to detect 92% of dengue hemorrhagic fever and 79.3% of dengue fever patients. Importantly, serum levels of galectin-9 were found to significantly correlate with the levels of several pro-inflammatory mediators, such as IL-1α, IL-8, IFN-γ-inducible protein 10, and vascular endothelial growth factor, during the critical phase of infection. Even during the recovery phase, there is a significant association between galectin-9 and several other DENV infection-induced mediators such as epidermal growth factor, IL-10, IL-8, and vascular endothelial growth factor [67]. Moreover, the knockdown of galectin-9 in dendritic cells results in the suppression of IL-12p40 production [63].

## 5. Targeting Galectin-9/Tim-3 in Treating Viral Infection

Given the broad-spectrum roles of Tim-3 and galectin-9 in regulating immune responses, the collected data indicate that targeting Tim-3 or galectin-9 may have a potential therapeutic use against viral infection [68]. The administration of Tim-3 Fc fusion protein significantly rescues a proportion of CD8^+^ T cells to produce IFN-γ and TNF-α when challenged with HBV peptides [59]. In addition, blocking Tim-3 rescues HBV-specific cytotoxic CD8^+^ T cell and IL-2-producing CD4^+^ T cell responses better than blocking PD ligand-1/2 [59]. Furthermore, blocking the galectin-9/Tim-3 interaction provides complementary effects to PD-1 inhibition in inducing HBV-specific T cells [59]. In response to influenza A virus challenge, blocking galectin-9 signaling by Tim-3 fusion protein treatment effectively induces and amplifies the intensity and quality of virus-specific CD8^+^ T cell responses in mice [16]. All together, these results suggest that disrupting the galectin-9/Tim-3 interaction can improve the immune response against viruses and may be a useful approach in developing therapeutics for HBV infection [69].

The treatment of HSV1-infected mice with MAbT25, a monoclonal antibody that induces the expansion of cells expressing the TNF receptor superfamily member 25 and the Tregs population. Recombinant galectin-9 significantly reduces the severity of stromal keratitis [70] by reducing the number of Th1 cells, reducing the total number of CD4^+^ T cells, upregulating anti-inflammatory cytokines, such as transforming growth factor-β and IL-10, and downregulating pro-inflammatory cytokines, such as IFN-γ and IL-6 [70]. The administration of galectin-9 to mice with HSV-induced Behçet’s disease inhibits pro-inflammatory cytokine production, attenuates inflammation, and reduces disease severity; however, galectin-9 administration also increases the number of Tregs [71]. Using a gene delivery approach, the introduction of a recombinant adenovirus 9-galectin-9 adenoviral plasmid intra-nasally into respiratory syncytial virus-infected mice results in the significant reduction of viral load, mucus secretion, and lung pathology [72]. Investigating the underlying mechanisms reveals several possible contributing factors such as the inhibition of Th17 cell production, induction of Tregs expansion, and regulation of CD8^+^ T cell apoptosis [72].

The interaction between soluble galectin-9 and Tim-3 expressed on the surface of activated CD4^+^ T cells results in the reduction of HIV-1 coreceptor expression and renders these cells less susceptible to HIV-1 infection and replication [73]. Because Tim-3 is consistently expressed on dysfunctional T cells during chronic HIV infection, recombinant galectin-9 administration provides another therapeutic option for HIV infection. Treatment with either anti-human Tim-3 or anti-human galectin-9 mAb suppresses bacterial colony forming units in co-cultures of monocyte-derived macrophages infected by mycobacterium tuberculosis and T cells from HIV-infected patients [23]. Given the high risk of mycobacterium tuberculosis infection in HIV-infected patients, especially in endemic areas, the results suggest the beneficial effects of inhibiting galectin-9/Tim-3 interaction in controlling mycobacterium tuberculosis growth in HIV-infected patients with co-infection by mycobacterium tuberculosis. Furthermore, recombinant galectin-9 treatment reverses HIV latency in vitro in the J-Lat HIV latency model [61]. In ex vivo experiments, the same group of researchers also demonstrated that recombinant galectin-9 reverses HIV latency in primary CD4^+^ T cells from HIV-infected and antiretroviral therapy-suppressed individuals with a potency stronger than vorinostat, a compound that inhibits histone deacetylase. Moreover, combining recombinant galectin-9 and a bromodomain inhibitor JQ1 was reported to have synergistic antiviral activity [61]. The recombinant galectin-9 administration also induces the expression of several anti-HIV host restriction factors, reduces the infectivity of progeny virus, and importantly, minimizes the possibility of recovery of the HIV reservoir once latency is reversed [61].

## 6. Perspectives

Administering galectin-9 to induce cellular apoptosis is a well-accepted approach in cancer therapy [43]; however, the opportunity to apply this concept for antiviral therapy remains to be investigated. The great challenge of this idea is that, in contrast to inducing apoptosis in cancer cells, causing cell death in virus-infected cells may result in severe tissue damage and thus is not favorable. According to structural analysis, different regions of galectin-9 may mediate different functions in terms of promoting Tregs or effector T cells activity. By introducing the appropriate galectin-9 component, we may be able to enhance the effector activity to fight against viruses or to reduce the generation of Tregs activity to increase immunity [55]. Along with the growing knowledge about this molecule, targeting galectin-9 may provide a direction of applying glycobiology knowledge for the treatment of viral infection. Furthermore, targeting galectin-9 may highlight a potential benefit for certain diseases, like DENV infection, for which the development of therapeutics has been challenging [74]. An additional advantage of applying galectin-9 for a therapeutic purpose is gained by the relatively clear pharmacokinetics of galectin-9 when introduced by the subcutaneous or intraperitoneal route in mice [44]. 

Overall, much remains to be understood about the galectin-9/Tim-3 signaling pathway. A previous study revealed that the galectin-9/Tim-3 interaction results in phosphorylation of the residues Y256 and Y263 in the Tim-3 tail and leads to the release of Bat3, thus initiating the Tim-3-mediated inhibitory signal [75]. The study also demonstrated that Bat3 can promote IFN-γ production and rendering Th1 cells resistant to galectin-9-mediated cell death [75]. Furthermore, Bat3 suppresses the development of T cell exhaustion, a phenomenon observed in chronic viral infection, and promotes the differentiation of effector T cells, which subsequently proliferate and produce IL-2 [75]. Because galectin-9 is a secreted and a resident intracellular molecule, additional studies investigating galectin-9-dependent intracellular signaling events may be helpful in delineating the different consequences generated in galectin-9-mediated effects. Given the complex consequences on immune responses following galectin-9/Tim-3 interaction, appropriate strategies targeting galectin-9 require more clear knowledge about the molecular mechanisms of galectin-9/Tim-3-mediated immune responses.
